# P-1801. Clinical Evaluation of a Novel, Extraction-free Isothermal Nucleic Acid Amplification Test for the Detection and Differentiation of HSV-1 and HSV-2 from Various Pediatric Sample Sources

**DOI:** 10.1093/ofid/ofaf695.1970

**Published:** 2026-01-11

**Authors:** Benjamin M Liu

**Affiliations:** Children's National Hospital /George Washington University, Washington, District of Columbia

## Abstract

**Background:**

HSV is a DNA virus that causes various infections in adults and children. While HSV-1 and HSV-2 share a preference for epithelial and neuronal tissues, HSV-1 usually causes cold sores and HSV-2 commonly leads to genital herpes or lesions. It is essential to rapidly and accurately detect and differentiate HSV-1 and -2 from various sample sources, especially from pediatric populations. However, there are significant diagnostic gaps in FDA-approved PCR tests for detection and differentiation of HSV-1 and -2, for example, limited approved sources, false positives and inter-type cross-reactivity. Herein we aimed to perform clinical evaluation of a novel, extraction-free isothermal nucleic acid amplification test (iAMP HSV 1/2, Atila BioSystems) for the detection and differentiation of HSV-1 and -2 from various pediatric sample sources.

**Methods:**

Swab samples collected from oral, skin, genital and eye sites of pediatric patients at Children’s National Hospital were detected using iAMP HSV 1/2 Detection Kit and standard-of-care Simplexa HSV 1 & 2 (Diasorin). Positive, negative and overall percentage agreement of iAMP test was determined against Simplexa test. Accuracy of type differentiation of iAMP HSV 1/2 Detection Kit was determined. Discrepant analysis by patient chart review was performed for any samples with discrepant results between both tests.

**Results:**

178 swab samples were collected from pediatric patients aged ranging from 1 month to 19 years. Among 104 Simplexa negative samples, 100.0% (104/104) were tested negative by iAMP test. Among 74 Simplexa positive samples, 96.0% (71/74) were tested positive by iAMP test, with 3 discrepant samples (Simplexa positive but iAMP negative). The accuracy of type differentiation of iAMP test was 100% (71/71) compared to Simplexa test. After discrepancy analysis, final positive, negative and overall percentage agreement of iAMP test were 98.6% (71/72), 100% (106/106), and 99.4% (177/178) compared to Simplexa test.

**Conclusion:**

This study demonstrated excellent clinical performance characteristics of iAMP HSV 1/2 Kit. It is a useful addition to a suite of molecular assays for detection and differentiation of HSV-1 and -2 from various pediatric sample sources and warrants further validation.

**Disclosures:**

All Authors: No reported disclosures

